# The role of chicken eggs in modulating sarcopenic obesity and gut microbiota in *db/db* mice

**DOI:** 10.3389/fmicb.2023.1281217

**Published:** 2023-10-19

**Authors:** Takuro Okamura, Yuka Hasegawa, Masahide Hamaguchi, Ryoichi Sasano, Michiaki Fukui

**Affiliations:** ^1^Department of Endocrinology and Metabolism, Graduate School of Medical Science, Kyoto Prefectural University of Medicine, Kyoto, Japan; ^2^AiSTI Science Co., Ltd., Wakayama, Japan

**Keywords:** egg, sarcopenic obesity, fecal microbiota transplantation, *Vampirovibrio*, gut microbiology

## Abstract

**Background:**

Sarcopenia obesity, in which loss of muscle mass and fat accumulation occur simultaneously, is the pathological basis of type 2 diabetes mellitus. The usefulness of chicken eggs in sarcopenia prevention has been reported in several previous studies. The purpose of this study was to determine the beneficial effects of chicken eggs in the prevention of sarcopenic obesity in *db/db* mice.

**Methods:**

We raised 8-week-old *db/db* male mice, a model of sarcopenia obesity, for 8 weeks and fed them a diet mixed with dried whole eggs. The fecal microbiota transplant (FMT) group was treated with antibiotics for 2 weeks, starting at 6 weeks of age, followed by FMT twice a week until 16 weeks of age.

**Results:**

Eggs administered to *db/db* mice showed increased grip strength (*p* = 0.022) and muscle mass (*p* = 0.013), decreased visceral fat mass (*p* = 0.005), and significantly increased physical activity (*p* < 0.001). The FMT group of egg-fed mice showed a significant improvement in glucose intolerance and sarcopenic obesity. Sequencing of gene expression in the small intestine showed that the gene expression of amino acid transporters such as *Slc6a18*, *Slc6a19*, and *Slc38a6* was increased in egg-fed mice. 16S rRNA sequencing of the gut microbiota showed an increase in the genus *Vampirovibrio* in both the egg-fed and FMT groups compared to that in egg-fed mice.

**Conclusion:**

The results of this study indicate that egg consumption not only increases the intake of amino acids and other nutrients but also alters the intestinal microbiota and increases amino acid absorption from the intestinal tract, suggesting that eggs might contribute to the ameliorative mechanism of sarcopenic obesity.

## Graphical abstract

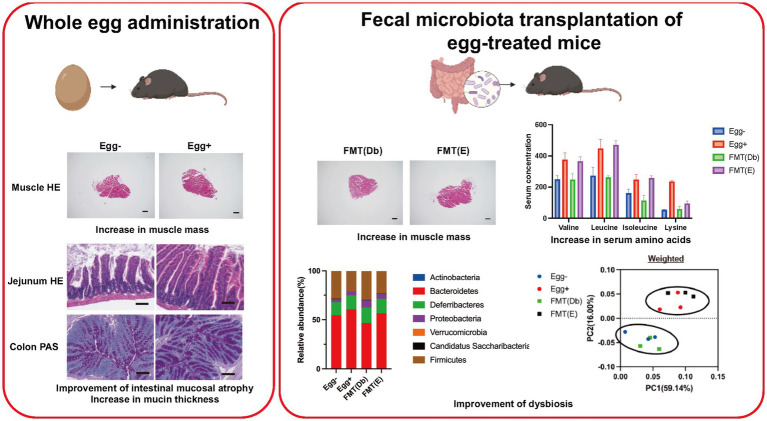

## Introduction

The escalating global incidence of type 2 diabetes is incontrovertible (IDF Diabetes Atlas | Tenth Edition, 2022). The primary etiological factors for type 2 diabetes are hyperalimentation and sedentary lifestyles (Van Strien and Van de Laar, 2008; Global Strategy on Diet, Physical Activity and Health - 2004, 2022). The fundamental pathophysiological underpinnings of this ailment are concomitant augmentation in visceral adiposity and diminution in muscularity, termed sarcopenic obesity (Mesinovic et al., 2019). In previous investigations, we delineated several studies in both human and animal models of sarcopenic obesity and type 2 diabetes, and discovered that leptin receptor-deficient *db/db* mice, marked by hyperphagia and physical inactivity, manifest sarcopenic obesity coupled with dysbiosis (Okamura et al., 2019). Furthermore, administering propolis or sodium/glucose cotransporter 2 inhibitors to these specimens not only mitigates sarcopenic obesity, but also ameliorates glucose dysregulation, concomitant with an enhancement in locomotor activity and muscle hypertrophy (Bamba et al., 2022; Okamura et al., 2022). Thus, circumventing sarcopenic obesity is paramount for averting the initiation and exacerbation of type 2 diabetes.

Dietary modulation is universally advocated as a prophylactic strategy for sarcopenia (Morley et al., 2014). Both whole eggs and egg albumen have been used as efficacious dietary countermeasures, with empirical evidence underscoring their beneficial effects on muscle hypertrophy and functional preservation (Van Vliet et al., 2017; Bagheri et al., 2020, 2021). Owing to their commendable amino acid composition, whole eggs, including their yolk, are considered a quintessential protein reservoir (Wilson and Wilson, 2006), furnishing 154 kcal and 12.3 g of protein (Bagheri et al., 2020). A study by Van Vliet et al. showed that post-exercise consumption of whole eggs in resistance-trained males augmented the phosphorylation of specific protein kinases within the mTOR cascade, culminating in more pronounced post-exertional myofibrillar protein synthesis compared to ingestion of egg albumen alone (Van Vliet et al., 2017). In fact, In our distinct cohort analysis, we selected a random assortment of 33 geriatric males with type 2 diabetes, each consuming in excess of 60 g of eggs daily, and juxtaposed their Skeletal Muscle mass Index (SMI) against a comparable cohort consuming less than the stipulated quantity (Supplementary Figure S1).

The interplay between sarcopenia and gut microbiota composition has been delineated in an array of studies (Siddharth et al., 2017). For example, probiotic supplementation with *Lactobacillus acidophilus* and *Bifidobacterium bifidum* markedly enhanced muscle mass, strength, and endurance of senescent mice (Ni et al., 2019). While myriad animal studies have expounded the metabolic ramifications of egg supplementation, the outcomes are multifactorial, encompassing not only direct nutritional benefits, but also intricate alterations in gut microbiota dynamics (Jia et al., 2022). However, the literature remains bereft of any examination of the effect of egg-induced fecal microbiota transplantation (FMT) on muscularity.

The objective of this study was to elucidate the potential anti-sarcopenic obesity attributes of egg supplementation using *db/db* mice as a representative model of sarcopenic obesity.

## Methods

### Human investigation

We have embarked on an ongoing longitudinal cohort study, KAMOGAWA-DM, since 2014 (Sakai et al., 2018). The study methodology adhered rigorously to the tenets of the Declaration of Helsinki. Ethical clearance was secured from the Institutional Review Board of Kyoto Prefectural University of Medicine (RBMR-E-466), and all participants provided written informed consent. For this investigation, we selected participants who underwent peripheral blood collection. The cohort predominantly comprised Japanese KAMOGAWA-DM participants, all of whom were ambulatory and enrolled from the outpatient clinics of the Kyoto Prefectural University of Medicine between August 2015 and September 2022. Dairy consumption was quantified using a brief self-administered Diet History Questionnaire.

### Murine model

Ethical approval for all experimental protocols was obtained from the Animal Care Committee of Kyoto Prefectural University of Medicine (Approval No. M2022-82). We performed a randomized clinician-masked study of male *db/db* mice.

#### Phase I

**Figure 1:**
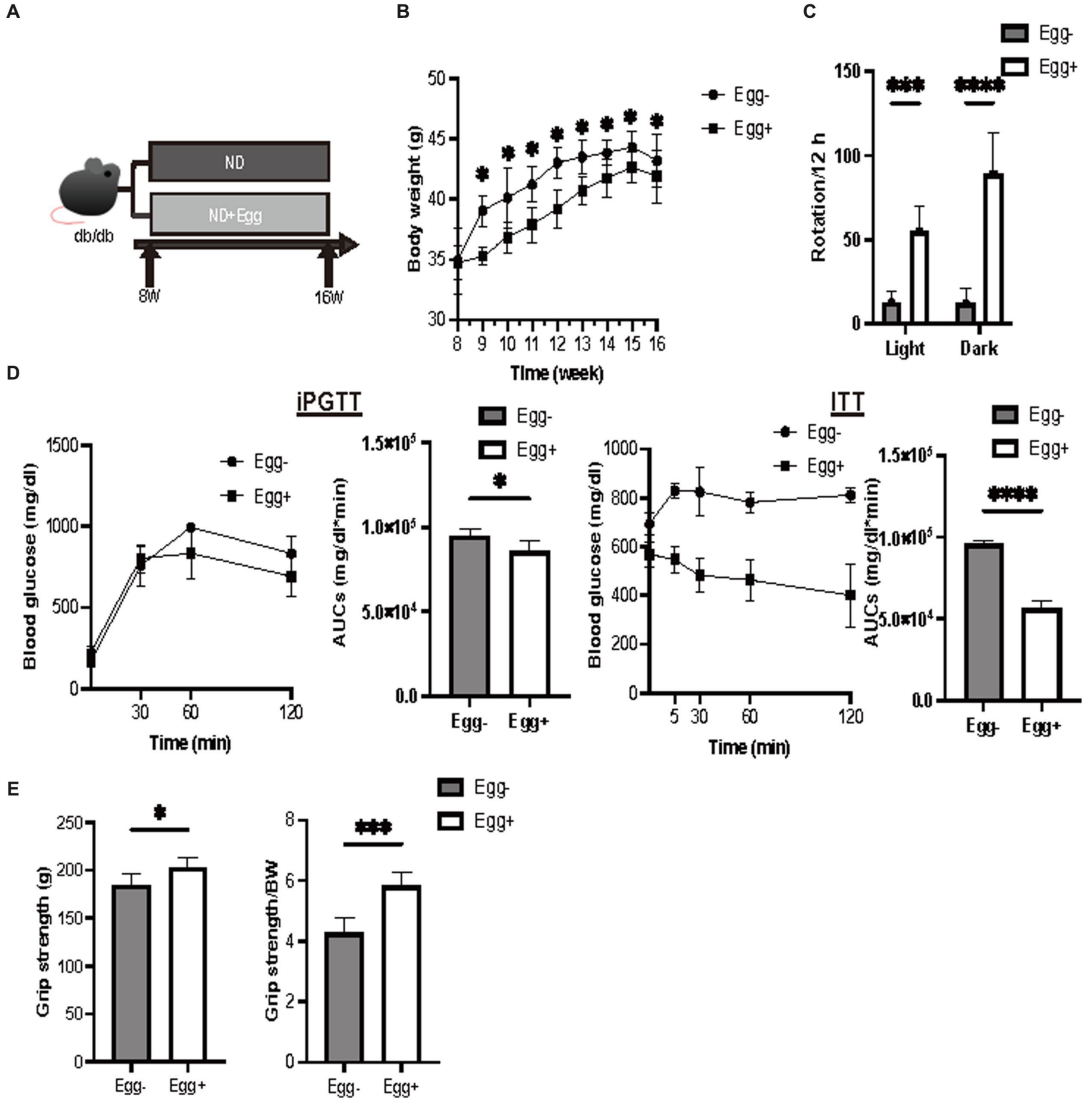
Grip strength was higher in the egg-fed mice. **(A)** Scheme of experimental study design. Supplementation of dried whole eggs began at 8 weeks of age, and mice were sacrificed at 16 weeks of age. **(B)** Body weight changes in mice (*n* = 6). **(C)** Rotation numbers in the light and dark periods measured by the running wheel (*n* = 6). **(D)** Results and area under the curve (AUC) analysis of intraperitoneal glucose tolerance test (2 g/kg body weight) in 15-week-old mice (*n* = 6). Results and AUC analyses of insulin tolerance test (0.5 U/kg body weight) for 15-weeks-old mice (*n* = 6). **(E)** Grip strength, absolute and relative (*n* = 6). Data are represented as the mean ± SD values. Data were analyzed using Welch’s *t*-tests. *p < 0.05, ***p < 0.001, and ****p < 0.0001.

We procured 7-week-old db/db male mice were procured from Shimizu Laboratory Materials (Kyoto, Japan) and reared under stringent aseptic conditions. Isogenic mice born in the aforementioned facility were designated for the experiments. Mice were individually caged and administered a standard diet (ND; 345 kcal/100 g, fat kcal 4.6%; CLEA, Tokyo, Japan) for 8-week duration commencing at 8 weeks of age. The feed portions are evenly distributed. Sample size estimation utilized EZR to target relative grip strength metrics. Based on the derived parameters, the requisite sample size was determined as six. Consequently, 12 mice were bifurcated into two distinct cohorts: (1) those devoid of dehydrated whole egg supplementation and (2) those receiving dehydrated whole eggs (1 weight %, Kewpie Corporation, Tokyo, Japan). Given our empirical findings that elderly type 2 diabetic patients consuming an excess of 60 g/day of eggs manifested reduced sarcopenia prevalence (Supplementary Figure S1), we incorporated dehydrated whole eggs into the feed at 1 weight percent. This ensured minimum ingestion of 40 mg/day of eggs by the mice, extrapolating to a daily feed rate of 4–6 g/day. After overnight fasting, the mice were euthanized at 16 weeks of age. The absolute and relative masses of various muscles and adipose tissues were recorded (Figure 1A).

#### Phase II

FMT is a widely accepted approach to elucidate the etiopathogenic role of the gut microbiota in models of disease related to the gut microbiota. To achieve better engraftment, depletion of the recipient gut microbiota using antibiotics (ampicillin, neomycin, metronidazole: 1 g/L each; vancomycin: 0.5 g/L; 200 μL/day by oral-gastric gavage) was performed for 2 weeks from 6 to 8 weeks of age prior to FMT (S1). After 3 days of recovery, FMT was performed twice weekly. Briefly, 200–300 mg of fresh stool was collected from 16-week-old *db/db* mice fed without egg and from those fed egg. The stool was homogenized in 5 mL of phosphate-buffered saline (PBS) and sedimented under gravity for 2 min; 200 μL of the supernatant, thus obtained, was administered to each receiving mouse (S2) and used for extraction of the gut. Body weight changes, glucose tolerance, and grip strength were evaluated for *db/db* mice subjected to FMT of stool from 16-week-old *db/db* mice fed without egg [FMT(db)] and from *db/db* mice fed egg [FMT(E)]. The mice were then sacrificed, and absolute and relative soleus and plantaris muscle mass and epididymal fat mass were determined. At 16-weeks of age, after overnight fasting, all mice were killed by administering a combination of anesthetics.

### Locomotor activity assessment

Mice were individually housed and equipped with a running wheel apparatus (MK-713; Muromachi Kikai). Rotational counts of the wheel were logged for each nocturnal 12-h cycle.

### Glucose metabolism and analytical protocols

Intraperitoneal glucose tolerance tests (iPGTT) and insulin tolerance tests (ITT) were performed on 15-week-old mice. Blood glucose metrics were measured using a glucometer (Gultest Mint II; Sanwa Kagaku Kenkyusho, Nagoya, Japan). The cumulative glucose responses were integrated to compute the area under the curve (AUC).

### Grip strength quantification

Grip dynamometry was undertaken using a dedicated meter for murine models (model DS2-50 N, IMADA Co., Ltd., Toyohashi, Japan). Sequential measurements were undertaken at regular intervals, and examiners remained unaware of the murine groupings. Grip strength was subsequently normalized to body mass.

### Histomorphometric analysis of skeletal muscle

The soleus muscle was chosen to evaluate myofiber cross-sectional areas. Staining and imaging protocols were deployed, and quantifications were performed using a BZ-X710 fluorescence microscope (Keyence, Osaka, Japan).

### Muscular gene expression profiling

The plantaris muscle was adopted to evaluate the gene expression in the muscles. The plantaris muscle of mice fasted for 16 h was excised and immediately frozen in liquid nitrogen. The samples were homogenized in ice-cold QIAzol Lysis reagent (Qiagen, Venlo, Netherlands) at 4,000 rpm for 2 min in a ball mill, and total RNA was extracted according to the manufacturer’s instructions. A High-Capacity cDNA Reverse Transcription Kit (Applied Biosystems, Foster City, CA, United States) was used to reverse transcribe the total RNA (0.5 μg) to first-strand cDNA, according to the manufacturer’s instructions. The mRNA expression of *Foxo1*, *Mstn*, *Fbxo32*, and *Trim63* in the plantaris muscle was quantified using real-time reverse transcription-polymerase chain reaction (RT-PCR); TaqMan Fast Advanced Master Mix (Applied Biosystems) was used according to the manufacturer’s instructions. The PCR conditions were as follows: one cycle of 2 min at 50°C and 20 s at 95°C, followed by 40 cycles of 1 s at 95°C and 20 s at 60°C. The relative expression of each target gene was normalized to the *GAPDH* threshold cycle (Ct) values and quantified using the comparative threshold cycle 2^−∆∆Ct^ method. Signals from the ND-fed mice were assigned a relative value of 1.0. Expression levels in six mice from each group were determined, and RT-PCR was performed in triplicate for each sample (*n* = 6).

### Proteomic analysis via Western blotting

Gastrocnemius muscle was used to assess intramuscular proteins. Gastrocnemius muscle extracts were prepared in a radioimmunoprecipitation assay buffer (RIPA, ATTO, Tokyo; 50 mmol/L Tris (pH 8.0), 150 mmol/L NaCl, 0.5% deoxycholate, 0.1% SDS and 1.0% NP-40) containing a protease inhibitor cocktail (BioVision, Milpitas, CA, United States). Protein assays were performed using a BSA protein assay kit (Pierce/Thermo Scientific) in accordance with the manufacturer’s instructions. Total protein (40 mg) was electrophoresed in 15% SDS-PAGE gels. SDS–PAGE was performed using e-PAGEL (E-T/R15L; ATTO Corporation, Tokyo, Japan) and p-PAGEL slab gels (P–T16.5S; ATTO Corporation) according to the manufacturer’s instructions. Western blotting was carried out using standard protocols and proteins detected by ChemiDoc MP Imaging System (Bio-Rad Laboratories, Hercules, CA, United States). The signals were analyzed using Image Lab software (Bio-Rad). The fold change was calculated as the ratio between the optical density of each protein divided by Gapdh. First, 40–60 μg of protein extraction were incubated with the following primary antibodies; Akt (1:1,500), p-Akt (1:1,000), mTOR (1:1,500), p-mTOR (1:1,000), p70S6K1 (1:1,000), 4EBP1 (1:1,000), Myogenin (1:1,000), MyoD (1:1,000), Myosin Heavy Chain (MHC) (1:1,000), AMPKα (1:1,000), Foxo1 (1:1,000), and MuRF1 (1:1,000) or Gapdh (1:1,500) diluted with EzBlock Chemi (ATTO, Osaka, Japan) overnight at 4°C, followed by incubation with goat anti-mouse IgG secondary antibodies conjugated to horseradish peroxidase diluted with EzBlock Chemi for 60 min at room temperature. All the antibodies listed in this section were obtained from Santa Cruz Biotechnology (Santa Cruz, CA, United States).

### Histological examination of intestinal segments

The jejunum and colon were employed for pathological evaluation of the small and large intestine. Jejunum and colon removed from mice were immediately fixed in 10% buffered formaldehyde for 24 h at 22°C, embedded in paraffin, cut into 4 μm-thick sections, and stained with HE and periodic acid Schiff (PAS) stain in Carnoy’s solution. Images of the stained sections were captured using a fluorescence microscope (BZ-X710; Keyence). The villus height/width and crypt depth were estimated using the HE-stained sections at five locations per slide for each group of 10 animals with the ImageJ software (Version 1.53 k, NIH, Bethesda, MD, United States). Mucin grains and goblet cells (PAS^+^) were enumerated and reported as the average number of goblet cells (PAS^+^) per 10 crypts using the ImageJ software, as reported previously (Motta et al., 2015).

### Jejunal mRNA sequencing

The jejunum of the mice was excised and immediately frozen in liquid nitrogen. The RNA extraction method was the same as that for the muscle described in the section, “Gene expression analysis in murine muscle.” Gene set enrichment analysis (GSEA) was performed using GSEA desktop software (Subramanian et al., 2005). Gene set permutations were repeated 1,000 times to obtain a normalized enrichment score, and a cutoff value of p of 0.05 was used to filter out significant enrichment results. Visualization of global mRNA expression associated with amino acids, fatty acids, and glucose transporters was done using volcano plots and heat maps.

### Measurement of lysine levels in the serum and skeletal muscle samples

Lysine levels in murine sera and gastrocnemius muscle were determined using gas chromatography–mass spectrometry (GC/MS). Methods are described in detail in Supplementary material.

### 16S rRNA sequencing of fecal samples

Appendicular feces were used for gut microbiota analysis. The Kyoto Encyclopedia of Genes and Genomes (KEGG) ortholog abundance predictions were obtained using the Phylogenetic Investigation of Communities by Reconstruction of Unobserved States (PICRUSt2) software (Douglas et al., 2020).

The relative abundance of phyla in the groups was evaluated using one-way ANOVA with Holm–Šídák multiple-comparison test. Alpha diversity (defined as the diversity within an individual sample) was analyzed using the Chao1 (Chao et al., 2006), Shannon (Shannon, 1948), and Gini–Simpson indices (Simpson, 1949).

The relative abundance of bacterial genera between the groups was evaluated using linear discriminant analysis (LDA) coupled with effect size measurements (LEfSe) (accessed on May 15, 2022)^1^ (Segata et al., 2011). With a normalized relative abundance matrix, LEfSe showed taxa with significantly different abundance, and the effect size of the feature was estimated using LDA. A *p*-value threshold of 0.05 (Wilcoxon rank-sum test) and an effect size threshold of 2 were used for all biomarkers discussed in this study.

In addition, principal coordinate analysis (PCoA) was performed to determine the effectiveness of FMT, and nonhierarchical *K*-means cluster analysis was performed, with the number of clusters to be generated prespecified as 2, using the Tinn-R Gui version 1.19.4.7, R version 1.36 (Ding and He, 2004).

### Statistical paradigms

Data computations and graphical representations were executed using GraphPad Prism software (version 9.3.1; San Diego, CA, United States). Binary group comparisons leveraged Welch’s t-test, while multi-group analyses utilized one-way analysis of variance with Tukey’s post-hoc test. A *p*-value threshold of <0.05 was deemed statistically significant.

## Results

### Body weight, momentum, iPGTT and ITT, and grip strength in mice fed ND with or without egg

Body weight was statistically significantly lower in the dried whole egg group (Egg+) than in the control group (Egg−) from 9 weeks of age (Figure 1B). Locomotor activity increased in both light and dark phases in the Egg+ mice compared to the Egg− mice (Figure 1C). Evaluation of glucose tolerance showed a decrease in blood glucose in the Egg+ mice in both iPGTT and ITT (Figure 1D). Absolute and relative grip strength was also higher in the Egg+ mice, compared to the Egg− mice (Figure 1E).

### Assessment of skeletal muscle and expression of genes and proteins related with muscle atrophy

**Figure 2:**
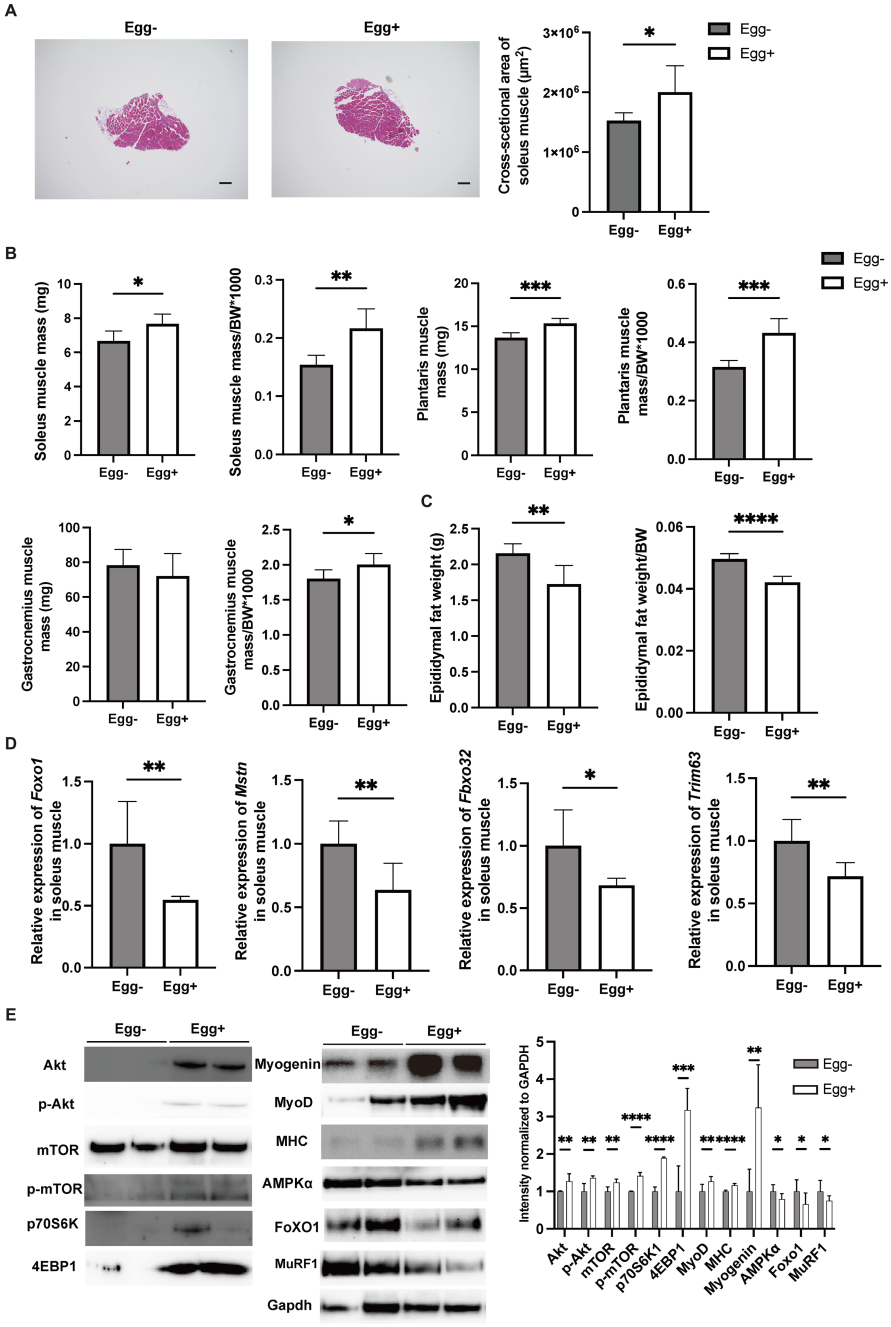
Egg-fed mice had higher skeletal muscle mass and lower gene expression associated with muscle atrophy. **(A)** Representative images of soleus muscle sections stained with hematoxylin & eosin (HE) for 16-weeks-old mice. The scale bar shows 100 μm. Cross-sectional area of the soleus muscle (*n* = 6). **(B)** Soleus muscle weight, absolute and relative (*n* = 6), plantaris muscle weight, absolute and relative (*n* = 6), and gastrocnemius muscle weight, absolute and relative (*n* = 6). **(C)** Epididymal fat weight, absolute and relative in 16-weeks-old mice (*n* = 6). **(D)** *Foxo1*, *Mstn*, *Fbxo32*, and *Trim63* mRNA expression in plantaris muscle normalized by *Gapdh* expression (*n* = 6). **(E)** Akt, mTOR, p70 S6K, 4E-BP1, Myogenin, MyoD, Myosin heavy chain (MHC), AMPKα, FoXO1, and MuRF1 in gastrocnemius muscle. Gapdh was used as a loading control. Intensity normalized to GAPDH was shown (*n* = 6). Data are represented as the mean ± SD values. Data were analyzed using Welch’s *t*-tests. *p < 0.05, **p < 0.01, ***p < 0.001, and ****p < 0.0001.

Skeletal muscles were also evaluated. The cross-sectional area of the soleus muscle was significantly higher in the egg+ mice (Figure 2A), and absolute and relative soleus and plantaris muscle weight of the Egg+ mice were higher than those of the Egg− mice (Figure 2B). While there was no clear difference in the absolute gastrocnemius muscle weight between the two groups, the relative weight was higher in the egg+ mice (Figure 2B). On the other hand, both the absolute and relative epididymal fat weight was lower in the Egg+ mice than in the Egg− mice (Figure 2C). Gene expression of *Foxo1*, *Mstn*, *Fbxo32*, and *Trim63*, which are genes related to muscle atrophy, was significantly lower in the Egg+ mice than in the Egg− mice as evaluated by RT-PCR (Figure 2D). We examined the protein expression levels in skeletal muscle. Activation of Akt/mTOR signaling was detected by Western blotting (Figure 2E). Furthermore, we found activation of downstream effector molecules (p70 S6K and 4E-BP1) and signals of MyoD, Myogenin, and MHC involved in muscle synthesis, while AMPKα, FoXO1, and MuRF1 signals were inactivated in Egg+ mice.

### Histological analysis of the jejunum and colon, and fold change in the expression of transporters in mice fed with and without egg

**Figure 3:**
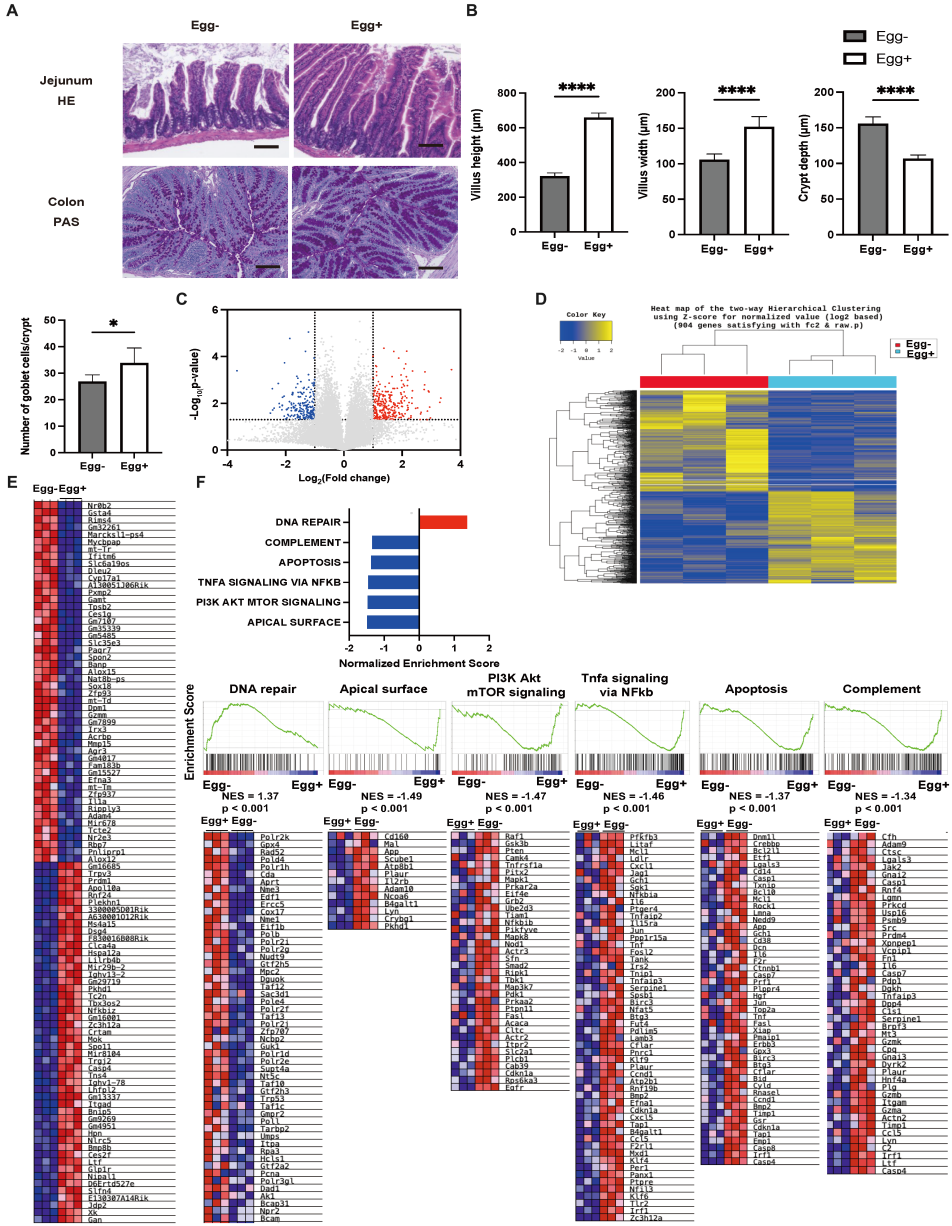
Inflammation of the small intestine was significantly improved in egg-fed mice. **(A)** Representative images of jejunum stained with hematoxylin and eosin (HE) and colon stained with periodic acid Schiff (PAS) for 16-weeks-old mice. The scale bar shows 100 μm. **(B)** Height and width of villus, and depth of crypt (*n* = 6). Number of goblet cells/crypt (*n* = 6). **(C)** Volcano plot of global mRNA expression level of two groups (*n* = 3). *Red*, FC > 2 and *p* < 0.05; *Blue*, FC < 2 and *p* < 0.05. **(D)** Hierarchical clustering analysis (*n* = 3). **(E)** Heatmap of upregulated and downregulated 50 genes between two groups (*n* = 3). **(F)** Core enriched gene normalized enrichment scores (NES) were established with GSEA using the DEG gene set (*n* = 3). Enrichment plots and heat maps of core enrichment genes were generated by GSEA using the DEG gene sets. NES and nominal *p*-values are shown for each gene set (*n* = 3). Data are represented as the mean ± SD values. Data were analyzed using Welch’s *t*-tests. *p < 0.05 and ****p < 0.0001.

Next, the small and large intestines were evaluated (Figure 3A). Both villus height and width were significantly higher in the Egg+ mice compared to the Egg− mice, while the depth of the crypts was lower. In addition, the number of goblet cells was significantly higher in the Egg+ mice (Figure 3B). In the mRNA sequencing of the small intestine, 284 genes were statistically significantly upregulated, and 202 genes were downregulated in Egg+ mice compared to Egg− mice (Figure 3C). Heatmap of the two-way hierarchical clustering analysis based on 984 genes were shown in Figure 3D. Heatmaps of the top 50 validated genes are then shown in Figure 3E. The heatmaps were generated by GSEA software and the differential expression of up-regulated (red) and down-regulated (blue) genes within each chip was scaled according to the color code depicted. The expression of *Gan*, *Xk*, *Jdp2*, *E130307a14rik*, and *Slfn4* was upregulated and that of *Nrob2*, *Gsta4*, *Rims4*, *Gm32261*, and *Marcksl1-ps4* was downregulated in Egg+ mice (Figure 3E). GSEA revealed that the hallmark DNA repair gene set was significantly upregulated and the hallmark apical surface, PI3K Akt mTOR signaling, Tnfa signaling via NFkb, apoptosis, and complement was downregulated in Egg+ mice (Figure 3F). In addition, gene ontology (GO) enrichment analysis was also performed. Multidimensional scaling analysis revealed similar genetic changes with egg consumption (Supplementary Figure S2A). Moreover, GO terms concerning “response to stimulus” in biological process, “binding” and “protein binding” in molecular function, and “cellular anatomical entity” in cellular component was upregulated in Egg+ mice (Supplementary Figure S2B).

**Figure 4:**
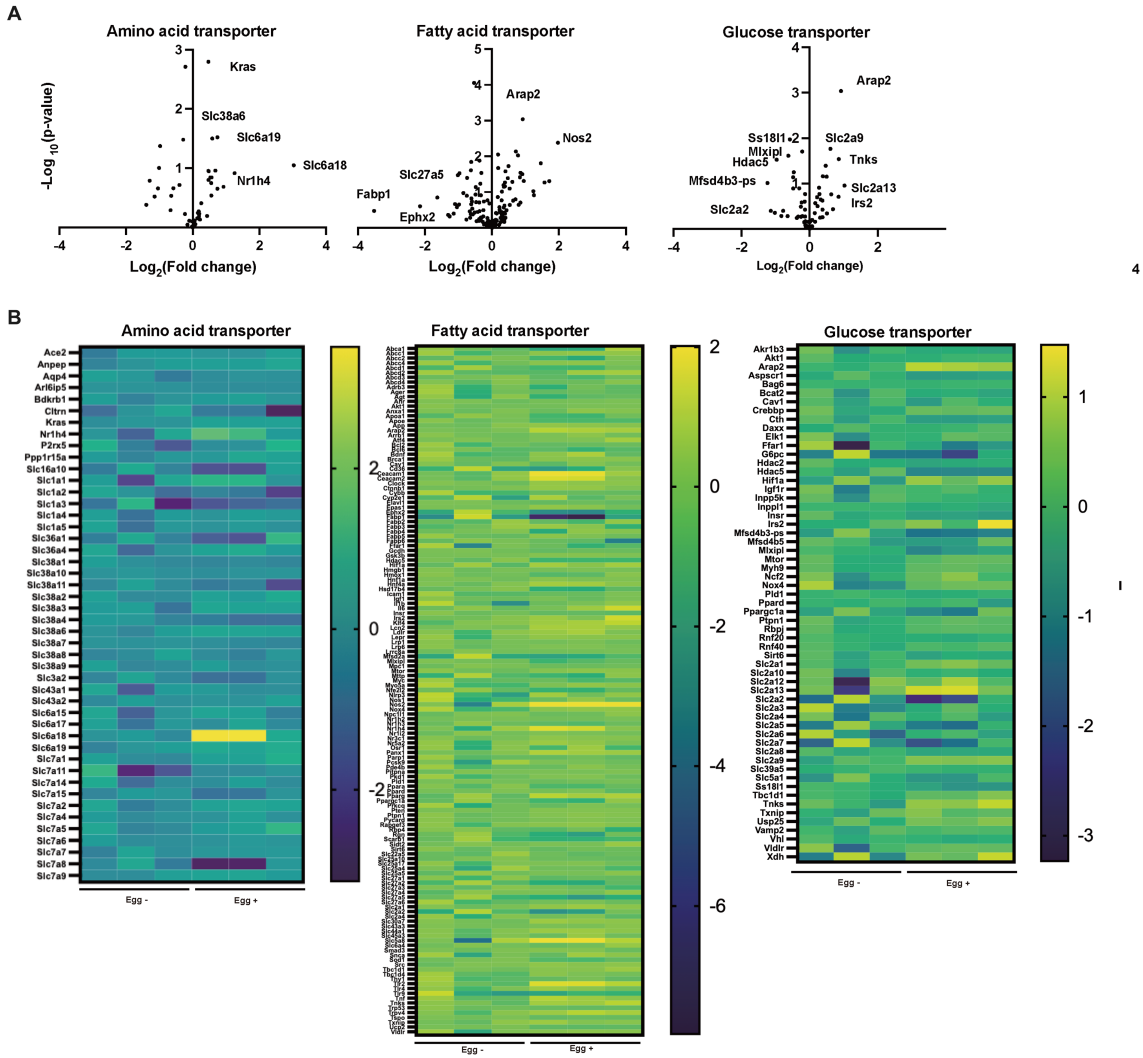
Supplementation of egg changed the gene expression related with nutrition transporter. **(A)** Volcano plots of amino acid transporter, fatty acid transporter, and glucose transporter (*n* = 3). **(B)** Heatmaps of amino acid transporter, fatty acid transporter, and glucose transporter (*n* = 3).

The gene expression of amino acid transporters such as *Slc6a18*, *Slc6a19*, and *Slc38a6* was upregulated in the Egg+ mice (Figures 4A,B). On the other hand, gene expression of fatty acid transporters, such as *Fabp1*, *Slc27a5*, and *Ephx2* was downregulated in the Egg+ mice and that of glucose transporter, such as *Slc2a2*, *Mfsd4b3-ps*, *Hdac5*, *Mixipl*, and *Ss18l1* was downregulated.

### Momentum, iPGTT and ITT, and grip strength in mice with fecal microbiota transplantation

**Figure 5:**
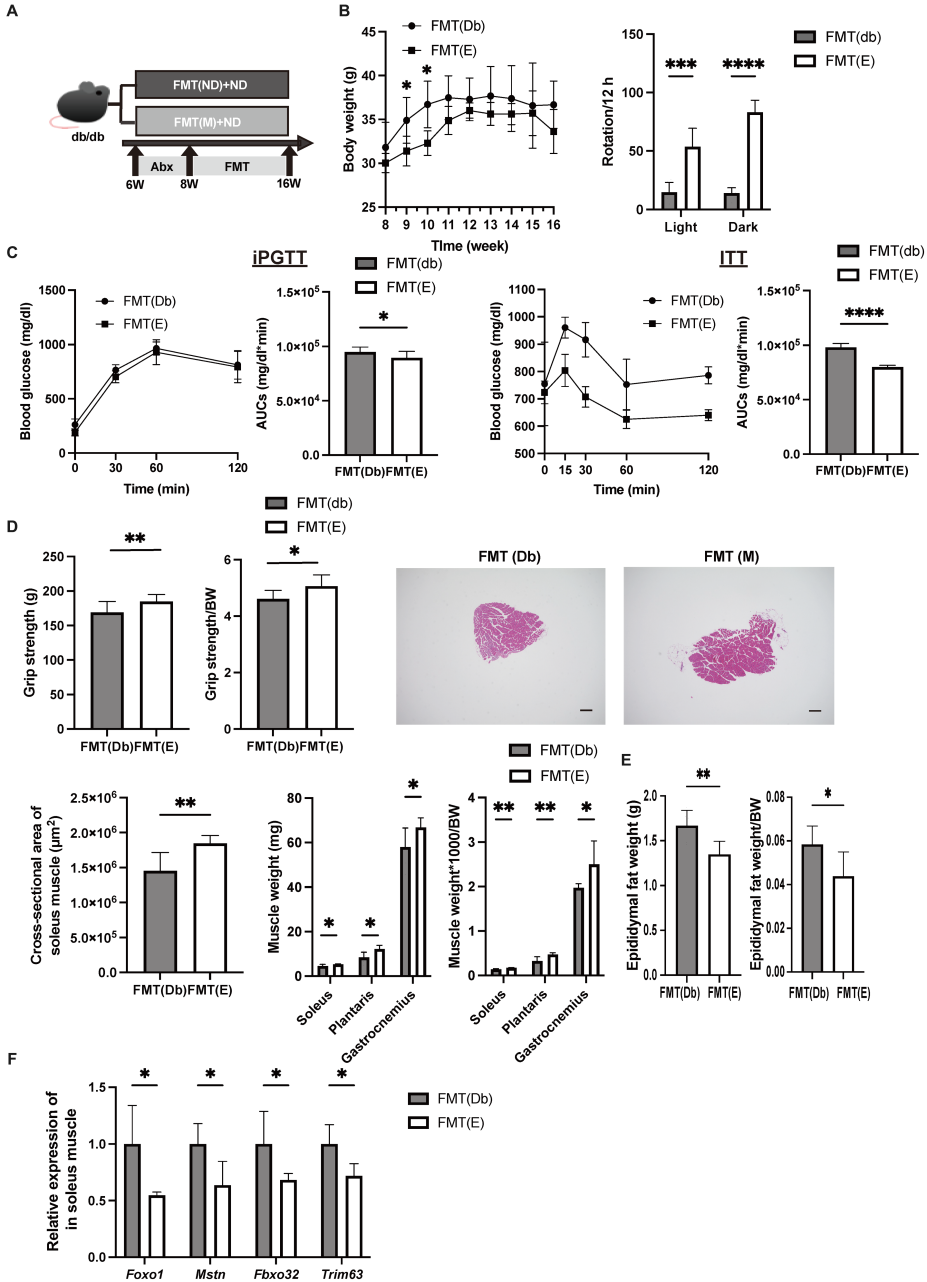
Skeletal muscle mass was higher and gene expression related with muscle atrophy was lower by fecal microbiota transplantation of *db/db* mice fed with egg. **(A)** Scheme of experimental study design. Antibiotic depletion of recipient gut microbiota was performed for 2 weeks from 6 to 8 weeks of age prior to fecal microbiota transplantation (FMT). **(B)** Body weight changes in mice (*n* = 6) and rotation numbers in the light and dark periods (*n* = 6). **(C)** Results and area under the curve (AUC) analysis of intraperitoneal glucose tolerance test in 15-week-old mice (*n* = 6). Results and AUC analyses of insulin tolerance test for 15-weeks-old mice (*n* = 6). **(D)** Grip strength, absolute and relative (*n* = 6), representative images of soleus muscle sections stained with hematoxylin and eosin (HE) for 16-weeks-old mice. (scale bar: 100 μm), cross-sectional area of soleus muscle, absolute and relative weight of soleus, plantaris, and gastrocnemius muscle, and absolute and relative epididymal fat weight (*n* = 6). **(F)** *Foxo1*, *Mstn*, *Fbxo32*, and *Trim63* mRNA expression in plantaris muscle normalized by *Gapdh* expression (*n* = 6). Data are represented as the mean ± SD values. Data were analyzed using Welch’s *t*-tests. *p < 0.05, **p < 0.01, ***p < 0.001, and ****p < 0.0001.

The same analysis as Egg− and Egg+ mice was performed in the FMT-treated group (Figure 5A). Body weight was lower in the FMT group of Egg+ mice [FMT(E)] than in the FMT group of Egg− mice [FMT(Db)], but only at 9 and 10 weeks of age (Figure 5B). Locomotor activity increased in both light and dark phases in the FMT(E) mice compared to the FMT(Db) mice (Figure 5B). Both iPGTT and ITT showed a decrease in blood glucose in the FMT(E) mice (Figure 5C). Absolute and relative grip strength were also higher in the FMT(E) mice, compared to those in the FMT(Db) mice (Figure 5D).

### Evaluation of soleus and plantaris muscles and expression of muscle atrophy genes in the FMT(Db) and FMT(E) mice

The cross-sectional area of the maximum bulge of the soleus muscle was significantly higher in the FMT(E) group (Figure 5D). Absolute and relative soleus, plantaris, and gastrocnemius muscles were higher in the FMT(E) mice than in the FMT(Db) mice (Figure 5D). Absolute and relative epididymal fat weight was lower in the FMT(E) mice compared to the FMT(Db) mice (Figure 5E). Gene expression of *Foxo1*, *Mstn*, *Fbxo32*, and *Trim63* was significantly lower in the FMT(E) mice compared to the FMT(Db) mice (Figure 5F).

### Amino acids concentration in serum and skeletal muscle

Valine, leucine, isoleucine, and valine concentrations in serum and skeletal muscle were higher in the Egg+ mice compared to the Egg− mice and in the FMT(E) mice compared to the FMT(Db) mice (Table 1).

**Table 1:** Concentration of amino acids in sera and skeletal muscle.

Amino acids		Egg−	Egg+	*p*-value	FMT(Db)	FMT(E)	*p*-value
Valine	Serum (μmol/L)	248.3 (23.3)	375.5 (43.4)	<0.0001	247.4 (37.3)	365.7 (28.5)	<0.0001
	Muscle (μmol/mg)	3.1 (0.9)	4.3 (0.4)	0.0028	1.5 (0.4)	3.7 (0.6)	<0.0001
Leucine	Serum (μmol/L)	272.2 (53.0)	447.1 (58.9)	<0.0001	261.4 (10.4)	469.6 (27.8)	<0.0001
	Muscle (μmol/mg)	1.6 (0.5)	2.4 (0.6)	0.0012	1.3 (0.0)	2.1 (0.1)	0.0017
Isoleucine	Serum (μmol/L)	160.1 (26.2)	247.2 (34.3)	<0.0001	113.3 (33.3)	257.8 (14.9)	<0.0001
	Muscle (μmol/mg)	1.4 (0.1)	1.6 (0.3)	0.2586	0.7 (0.1)	1.8 (0.2)	<0.0001
Lysine	Serum (μmol/L)	53.1 (2.3)	234.5 (6.2)	<0.0001	58.3 (19.4)	93.7 (15.9)	0.0003
	Muscle (μmol/mg)	2.0 (0.2)	3.3 (0.2)	<0.0001	1.6 (0.2)	2.4 (0.4)	<0.0001

Mean (SD). One-way ANOVA with Holm–Šídák multiple-comparison test was used to compare Egg− and Egg+ mice, FMT(Db), and FMT(E) mice.

### Fecal microbiota transplantation from mice donors fed egg to mice fed without egg

**Figure 6:**
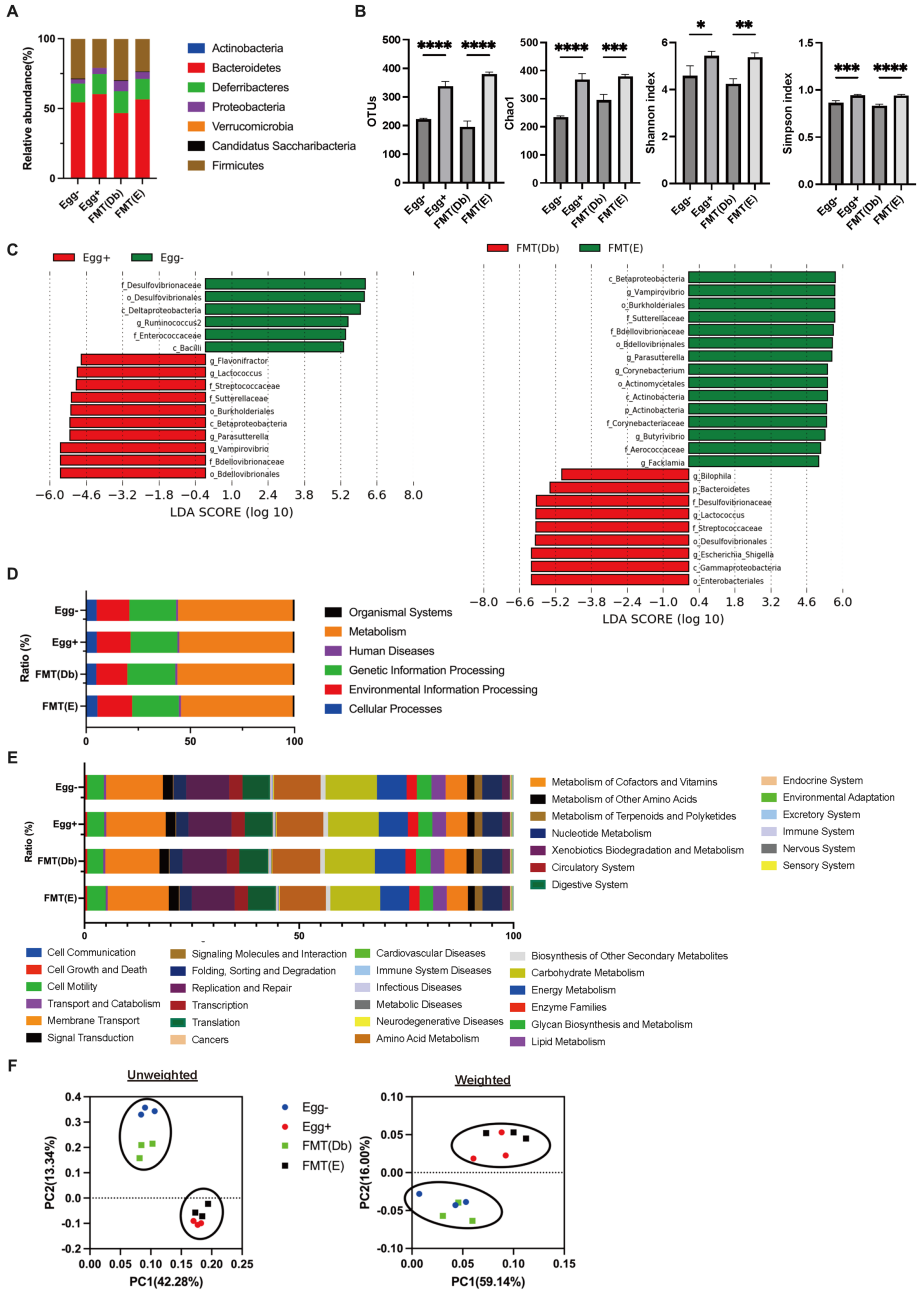
Changes in the gut microbiota by egg administration and fecal microbiota transplantation. **(A)** Relative gut microbiota abundance at the phylum levels (*n* = 3). **(B)** OTUs (*n* = 3), Caho1 (*n* = 3), Shannon-index (*n* = 3), and Simpson index (*n* = 3). **(C)** Egg− (green) and Egg+ (red) groups, and FMT (db) (green) and FMT **(E)** (red) groups’ LDA scores of gut microbiota. Description of KEGG pathway **(D)** class I and **(E)** class II (*n* = 3). **(F)** PCoA plots and k-means clustering for gut microbiota unweighted and weighted. Data are the mean ± SD values. Data were analyzed using one-way ANOVA with Holm-Šídák’s multiple-comparisons test. *p < 0.05, **p < 0.01, ***p < 0.001, and ****p < 0.0001.

In the gut microbiota analysis, we first examined the relative abundance of the different phylum in the four groups (Figure 6A). Compared to the control group, the Egg+ mice and FMT(E) mice had more phylum Bacteroidetes and fewer Firmicutes. OTUs, Chao1, Shannon index, and Simpson index, indices of diversity, were significantly higher in the Egg+ and FMT(E) mice compared to the control group, respectively (Figure 6B). Ten taxa (e.g., the genus *Vampirovibrio*, the genus *Parasutterella*, the genus *Lactococcus*, and the genus *Flavonifractor*) were overrepresented in the Egg+ mice and six taxa (e.g., the genus *RuminoCoccus2*) were underrepresented (Figure 6C). In addition, 15 taxa (e.g., the genus *Vampirovibrio*, the genus *Parasutterella*, the genus *Butyrivibrio*) were overexpressed and 5 taxa (e.g., the genus *Escheria_Shigella*, the genus *Lactococcus*, and the genus *Bilophila*) were underrepresented in the FMT(E) mice compared to the FMT(Db) mice (Figure 6C). Next, PICRUSt was used to analyze the differences in KEGG pathways of the gut microbiota between the Egg− and Egg+ mice and between the FMT (Db) and FMT (E) mice. In KEGG pathway class I, the organismal systems pathway was more prevalent in the Egg+ mice compared to the Egg− mice (Table 2; Figure 6D). In KEGG pathway class II, the amino acid metabolism, and metabolism of other amino acids pathway was suppressed in the Egg+ mice compared to the Egg− mice, and the human diseases pathway was suppressed in the FMT(E) mice compared to the FMT(Db) mice (Table 3; Figure 6E). Amino acid metabolism and metabolism of other amino acids pathways were increased in the Egg+ mice compared to the Egg− mice, and the same was true in the FMT(Db) mice. Finally, the efficacy of FMT was determined using PCoA. The results of unweighted and weighted clustering showed that the Egg− and FMT(Db) mice and the Egg+ and FMT(E) mice belonged to the same cluster (Figure 6F).

**Table 2:** KEGG pathway class I in four groups.

	Egg−	Egg+	*p*-value	FMT(Db)	FMT(E)	*p*-value
Cellular processes	5.04 (0.29)	5.08 (0.41)	0.8987	4.86 (0.70)	5.40 (0.26)	0.2553
Environmental information processing	15.68 (0.35)	16.22 (0.31)	0.1133	14.91 (1.11)	16.71 (0.32)	0.0540
Genetic information processing	22.45 (0.48)	22.51 (0.17)	0.8520	23.02 (0.41)	22.45 (0.33)	0.1301
Human diseases	0.94 (0.01)	0.90 (0.03)	0.1542	0.94 (0.01)	0.91 (0.01)	0.0252
Metabolism	55.00 (0.33)	54.43 (0.46)	0.1555	55.41 (1.38)	53.70 (0.08)	0.0993
Organismal systems	3.1 (0.9)	4.3 (0.4)	0.0099	0.86 (0.01)	0.84 (0.01)	0.0927

Mean (SD). One-way ANOVA with Holm–Šídák multiple-comparison test was used to compare Egg− and Egg+ mice, FMT(Db), and FMT(E) mice.

**Table 3:** KEGG pathway class II in four groups.

		Egg−	Egg+	*p*-value	FMT(Db)	FMT(E)	*p*-value
Cellular processes	Cell communication	0.00 (0.00)	0.00 (0.00)	–	0.00 (0.00)	0.00 (0.00)	–
	Cell growth and death	0.63 (0.02)	0.63 (0.02)	0.7014	0.65 (0.00)	0.63 (0.01)	0.039
	Cell motility	3.89 (0.32)	3.98 (0.43)	0.7974	3.72 (0.79)	4.33 (0.06)	0.2518
	Transport and catabolism	0.51 (0.07)	0.47 (0.03)	0.4468	0.50 (0.09)	0.44 (0.03)	0.3842
Environmental information processing	Membrane transport	13.17 (0.35)	13.78 (0.28)	0.0744	12.55 (0.89)	14.19 (0.30)	0.0389
	Signal transduction	2.30 (0.07)	2.24 (0.18)	0.5919	2.15 (0.23)	2.33 (0.02)	0.2704
	Signaling molecules and interaction	0.21 (0.00)	0.20 (0.00)	0.0647	0.21 (0.01)	0.19 (0.00)	0.0635
Genetic information processing	Folding, sorting, and degradation	2.89 (0.06)	2.83 (0.05)	0.3012	2.97 (0.08)	2.82 (0.05)	0.0414
	Replication and repair	10.07 (0.24)	10.09 (0.11)	0.9214	10.39 (0.29)	10.02 (0.15)	0.1233
	Transcription	3.10 (0.08)	3.09 (0.08)	0.9363	2.89 (0.12)	3.12 (0.03)	0.0367
	Translation	6.40 (0.26)	6.50 (0.04)	0.54	6.77 (0.17)	6.49 (0.15)	0.1019
Human diseases	Cancers	0.14 (0.00)	0.13 (0.01)	0.1148	0.14 (0.00)	0.13 (0.00)	0.2704
	Cardiovascular diseases	0.00 (0.00)	0.00 (0.00)	0.5504	0.00 (0.00)	0.00 (0.00)	0.5005
	Immune system diseases	0.04 (0.00)	0.04 (0.00)	0.7051	0.04 (0.01)	0.04 (0.00)	0.2443
	Infectious diseases	0.45 (0.01)	0.43 (0.02)	0.3631	0.46 (0.01)	0.44 (0.01)	0.0666
	Metabolic diseases	0.13 (0.00)	0.13 (0.00)	0.1692	0.13 (0.01)	0.12 (0.00)	0.1547
	Neurodegenerative diseases	0.17 (0.01)	0.17 (0.01)	0.7943	0.17 (0.00)	0.17 (0.00)	0.9292
Metabolism	Amino acid metabolism	10.88 (0.16)	10.93 (0.16)	0.0007	11.17 (0.33)	10.76 (0.03)	0.0063
	Biosynthesis of other secondary metabolites	1.16 (0.05)	1.11 (0.06)	0.334	1.10 (0.08)	1.06 (0.03)	0.4295
	Carbohydrate metabolism	12.01 (0.34)	11.83 (0.19)	0.4725	11.66 (0.17)	11.63 (0.10)	0.7888
	Energy metabolism	6.89 (0.10)	6.77 (0.14)	0.2662	7.12 (0.14)	6.77 (0.10)	0.0264
	Enzyme families	2.41 (0.07)	2.47 (0.08)	0.3707	2.46 (0.10)	2.43 (0.04)	0.6409
	Glycan biosynthesis and metabolism	3.40 (0.22)	3.25 (0.03)	0.307	3.43 (0.31)	3.14 (0.12)	0.2073
	Lipid metabolism	3.31 (0.06)	3.21 (0.02)	0.0484	3.19 (0.01)	3.17 (0.01)	0.1336
	Metabolism of cofactors and vitamins	5.03 (0.13)	4.97 (0.08)	0.5502	5.17 (0.05)	4.94 (0.05)	0.0045
	Metabolism of other amino acids	1.70 (0.02)	1.67 (0.05)	0.0083	1.69 (0.07)	1.62 (0.03)	0.0073
	Metabolism of terpenoids and polyketides	1.84 (0.06)	1.83 (0.04)	0.8209	1.91 (0.04)	1.80 (0.03)	0.018
	Nucleotide metabolism	4.59 (0.08)	4.61 (0.05)	0.7016	4.74 (0.15)	4.58 (0.03)	0.1394
	Xenobiotics biodegradation and metabolism	1.80 (0.00)	1.79 (0.00)	0.0153	1.75 (0.02)	1.80 (0.01)	0.01
Organismal systems	Circulatory system	0.00 (0.00)	0.00 (0.00)	0.1242	0.00 (0.00)	0.00 (0.00)	0.5005
	Digestive system	0.05 (0.00)	0.04 (0.00)	0.2474	0.04 (0.00)	0.04 (0.00)	0.0682
	Endocrine system	0.39 (0.01)	0.37 (0.01)	0.0441	0.38 (0.01)	0.37 (0.01)	0.3615
	Environmental adaptation	0.20 (0.02)	0.20 (0.01)	0.9286	0.19 (0.02)	0.20 (0.00)	0.5662
	Excretory system	0.03 (0.00)	0.02 (0.00)	0.3524	0.03 (0.01)	0.02 (0.00)	0.3509
	Immune system	0.11 (0.00)	0.11 (0.00)	0.0311	0.11 (0.00)	0.10 (0.00)	0.0249
	Nervous system	0.11 (0.00)	0.11 (0.01)	0.6154	0.11 (0.01)	0.11 (0.00)	0.3862
	Sensory system	0.00 (0.00)	0.00 (0.00)	–	0.00 (0.00)	0.00 (0.00)	–

Mean (SD). One-way ANOVA with Holm–Šídák multiple-comparison test was used to compare Egg− and Egg+ mice, FMT(Db), and FMT(E) mice.

## Discussion

In this study, the administration of desiccated whole eggs to a murine model of sarcopenic obesity led to increased muscle strength and mass, reduced visceral adipose tissue, and consequently, significantly improved locomotive capabilities. Furthermore, when fecal matter from dried whole egg-treated mice was transplanted into mice with sarcopenic obesity, the results mirrored those seen in the egg-treated group. These findings suggest that both nutritional enhancement and gut microbiota modulation through egg supplementation may play key roles in addressing sarcopenic obesity.

In the backdrop of the rising obesity crisis, a significant segment of the elderly population struggles with sarcopenic obesity. This condition is characterized by a notable decline in muscle mass accompanied by an accumulation of adipose tissue (Bosaeus and Rothenberg, 2016). Such physiological conditions can lead to anabolic resistance, which is often a result of insulin resistance and inflammation induced by adiposity (Guillet et al., 2012). Whole eggs, particularly their albumen, are highly regarded as effective nutritional interventions. They have been shown to preserve and even enhance muscle mass and function. In a study by Kim et al., 12 participants aged 57–74 were part of a crossover study where they consumed both an egg-based breakfast and an isonitrogenous cereal alternative. The results revealed that the egg-based diet significantly improved nitrogen balance, primarily due to reduced muscle protein breakdown (Kim et al., 2018). Furthermore, supplementing with egg whites for 3 weeks showed enhanced muscle strength in elderly female subjects compared to a maltodextrin placebo. This evidence underlines the capability of egg proteins to boost skeletal muscle health and delay the onset of sarcopenia in older individuals. This benefit arises from both reducing muscle protein breakdown and stimulating muscle protein synthesis. Studies involving rodents have also emphasized the potential benefits of specific amino acids in maintaining lean body composition. For example, rodents fed on a diet containing casein, albumin, and egg white proteins for 2 weeks showed significant muscle hypertrophy, especially in the soleus and extensor digitorum longus muscles. This hypertrophy was more pronounced after consuming egg whites than casein (Kido et al., 2022).

KEGG pathway analysis revealed an augmentation in the gut microbial communities associated with amino acid metabolism in both the desiccated whole egg and FMT(E) cohorts. Concurrently, mRNA sequencing of the jejunum ascertained significant upregulation in the expression of amino acid transporters *Slc6a18*, *Slc6a19*, and *Slc38a6* within the dried whole egg group. Such findings postulate that gut microbiota modulation via egg supplementation could potentially enhance skeletal muscle mass by fostering amino acid absorption in the jejunal region.

Moreover, both systemic and intramuscular levels of specific amino acids, notably valine, leucine, isoleucine, and lysine, manifested marked elevations in the cohorts administered with egg and FMT(E). Leucine, in particular, has been established to trigger muscle protein synthetic responses via activation of the mTORC1 signaling pathway (Crozier et al., 2005), Isoleucine has been shown to enhance the phosphorylation of 4E-BP1 and S6K1 In a distinct study, lysine supplementation in lagomorphs elicited an uptick in mRNA expression of MyoD and the protein expression of phosphorylated mTOR within skeletal muscle tissues (Liu et al., 2022). Lysine’s role in actuating the mTORC1 pathway in rodent skeletal muscle has been previously delineated (Sato et al., 2014). F Additionally, research has indicated that lysine can attenuate protein catabolism by facilitating the phosphorylation within the mTORC1 pathway in C2C12 myotubes (Sato et al., 2015). Consequently, it can be postulated that augmented lysine absorption, resultant from the administration of desiccated whole eggs, might have been instrumental in the observed enhancement of skeletal muscle mass.

Egg supplementation elicited discernible alterations in gut microbiota composition. Particularly, the genus *Vampirovibrio*, which exhibited an upsurge in both egg-fed and egg FMT rodents, has been previously associated with metabolic enhancements across diverse studies. For instance, this genus was found to be heightened in the intestines of obese mice subjected to bariatric surgery and showcased an inverse correlation with body weight and fasting glycemic levels (Liang et al., 2022). Taking into account the findings of the present investigation alongside prior research, it can be surmised that the genus *Vampirovibrio* might harbor protective attributes against obesity. Furthermore, the daily caloric intake from eggs in this study was a mere 0.2 kcal, suggesting that the observed enhancement in amino acid concentrations in blood and muscular tissues was more likely attributable to increased amino acid absorption mediated by gut microbiota alterations rather than solely to heightened protein intake from egg consumption.

In conclusion, this investigation elucidates that fecal transplantation from egg-treated mice led to a mitigation of sarcopenic obesity and improved glycemic profiles. It is postulated that both the augmented intake and absorption of amino acids, owing to modulations in the expression of amino acid transporter-associated genes within the jejunal epithelium, might be contributory to the observed improvements. Although this study provides invaluable insights into the potential health dividends of egg consumption, it is imperative to acknowledge its murine-centric focus. Thus, forthcoming clinical studies are requisite to validate these findings in human cohorts. It is anticipated that subsequent research endeavors will further embellish our comprehension of the health ramifications of egg consumption.

## Data availability statement

Derived data supporting the findings of this study are available from the corresponding author on request.

## Ethics statement

The animal study was approved by the Committee for Animal Research, Kyoto Prefectural University of Medicine. The study was conducted in accordance with the local legislation and institutional requirements.

## Author contributions

TO: Conceptualization, data curation, Investigation, Methodology, Writing – original draft. YH: Methodology, Writing – review & editing. MH: Writing – review & editing. RS: Methodology, Writing – review & editing. MF: Conceptualization, Writing – review & editing.

## Footnotes

^1^ http://huttenhower.sph.harvard.edu/lefse/
