# Supplementary material for: The role of chicken eggs in modulating sarcopenic obesity and gut microbiota in db/db mice
Source: Front Microbiol. 2023 Oct 19;14:1281217. doi: 10.3389/fmicb.2023.1281217 (PMC10622760; doi:10.3389/fmicb.2023.1281217)
Supplement: Supplementary file 1 [file Image_1.pdf]

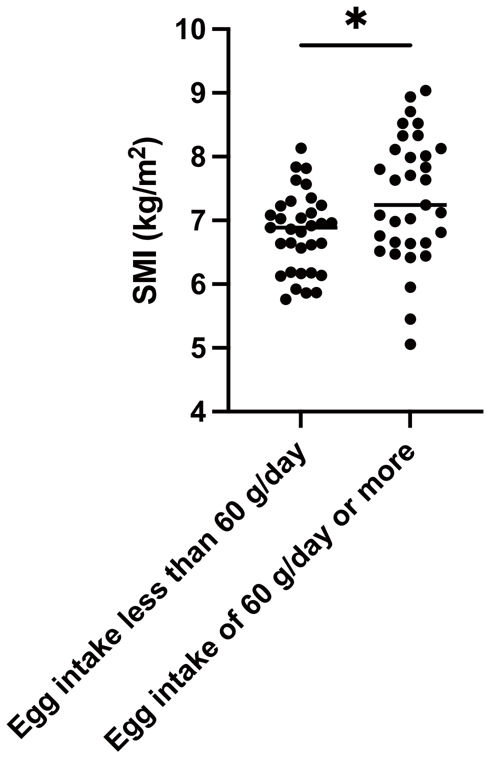

Supplementary figure 1. Differences in SMI by egg intake in elderly male patients with type 2 diabetes mellitus

Data are represented as the mean values. Data were analyzed using t-test. $p = 0.011$

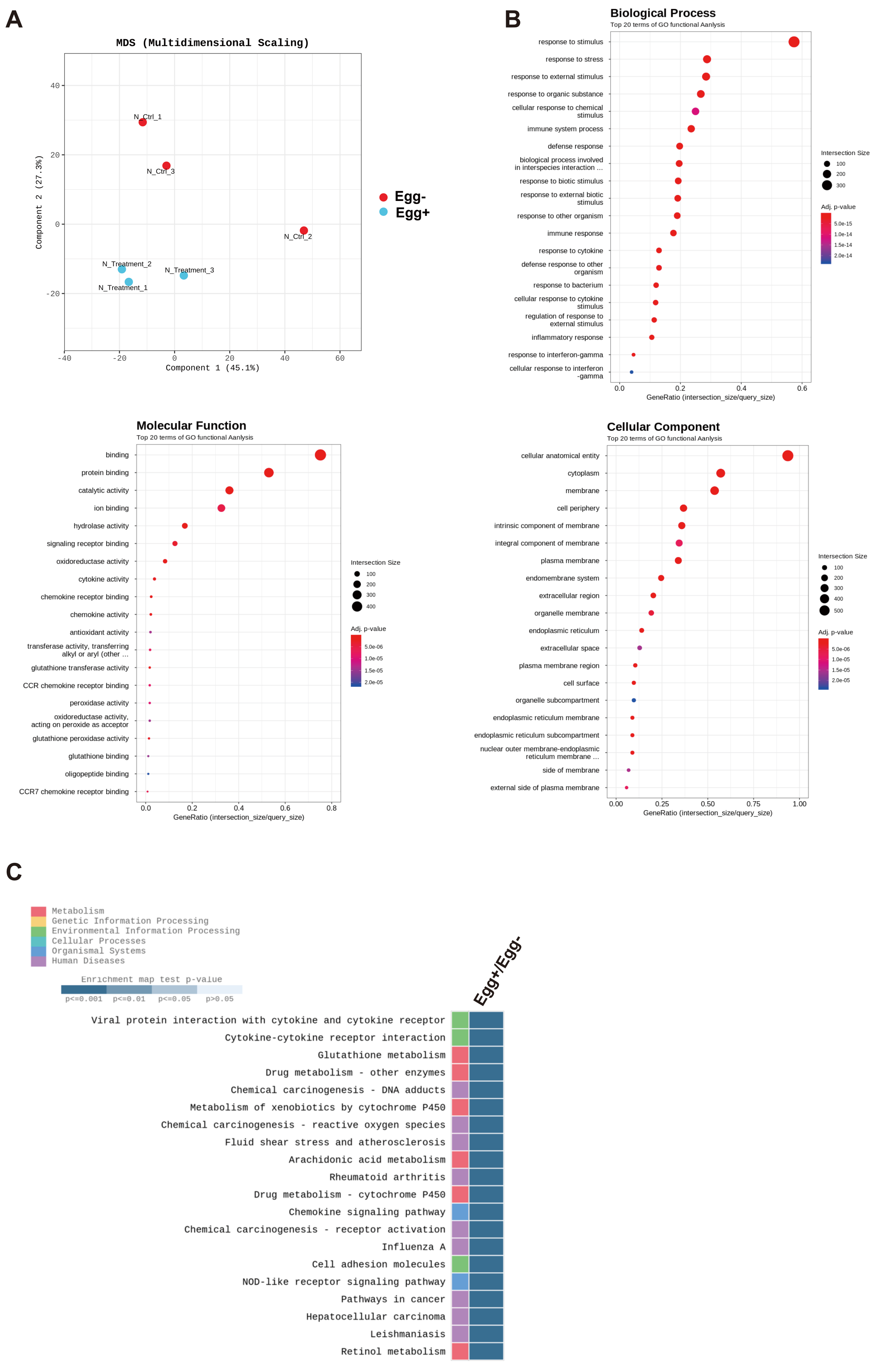

Supplementary figure 2. Differences in KEGG pathway by egg intake in elderly male patients with type 2 diabetes mellitus

(A) Multidimensional Scaling Analysis Red, Egg-; Blue, Egg+. (B) Top 20 terms of Gene Ontology Enrichment Analysis result were described by dot plot. Top 20 terms of Gene Oncology (GO) terms related to biological process, molecular function, and cellular component. (C) The heat map shows the results of the enrichment analysis for each pathway term. The gradient legend shows the enrichment raw p-value from a modified Fisher's exact test that determines the enrichment of each gene from the gene set. A raw p-value lower than 0.05 means that the pathway is significantly enriched.
